# What we can and cannot (yet) do with functional near infrared spectroscopy

**DOI:** 10.3389/fnins.2014.00117

**Published:** 2014-05-23

**Authors:** Megan Strait, Matthias Scheutz

**Affiliations:** Human-Robot Interaction Laboratory, Department of Computer Science, Tufts UniversityMedford, MA, USA

**Keywords:** functional near infrared spectroscopy, brain–computer interfaces, human–computer interaction, reliability, signal processing

## Abstract

Functional near infrared spectroscopy (NIRS) is a relatively new technique complimentary to EEG for the development of brain-computer interfaces (BCIs). NIRS-based systems for detecting various cognitive and affective states such as mental and emotional stress have already been demonstrated in a range of adaptive human–computer interaction (HCI) applications. However, before NIRS-BCIs can be used reliably in realistic HCI settings, substantial challenges oncerning signal processing and modeling must be addressed. Although many of those challenges have been identified previously, the solutions to overcome them remain scant. In this paper, we first review what can be currently done with NIRS, specifically, NIRS-based approaches to measuring cognitive and affective user states as well as demonstrations of passive NIRS-BCIs. We then discuss some of the primary challenges these systems would face if deployed in more realistic settings, including detection latencies and motion artifacts. Lastly, we investigate the effects of some of these challenges on signal reliability via a quantitative comparison of three NIRS models. The hope is that this paper will actively engage researchers to acilitate the advancement of NIRS as a more robust and useful tool to the BCI community.

## 1. Introduction

The primary aim of human–computer interaction (HCI) research is to develop methods and tools to facilitate effective interaction between people and with computer systems. While current modes of interaction mainly rely on tactile communication, there is a growing body of research on using brain-based sensors as an additional information channel (e.g., Tan and Nijholt, 2010; Zander and Kothe, 2011; Strait et al., 2014a). Socially-aware systems that can *capture and respond to* changes in anxiety, attention, arousal, and other user states have been found to be more effective in engaging people (e.g., Szafir and Mutlu, 2012). Hence, research on neurophysiological signals has been gaining the attention of researchers in human–computer interaction in recent years (e.g., Bainbridge et al., 2012; Frey et al., 2014; Strait and Scheutz, 2014).

Amongst this work, electroencephalography (EEG) is the most widely used technology in HCI, as it provides high temporal resolution and has general success in measuring a wide array of user states such as workload, attention, fatigue, and affect (Frey et al., 2014). However, EEG has limited spatial resolution, thus constraining its applicability for measuring region-specific brain activity. Conversely, high spatial resolution can be achieved using fMRI, but at a cost to both participant mobility and temporal resolution (e.g., Canning and Scheutz, 2013; Frey et al., 2014). Hence, functional near infrared spectroscopy (NIRS; also referred to as fNIRS or fNIR) is a promising alternative, achieving some middle ground in spatial and temporal resolution as well as mobility between the EEG and fMRI technologies (e.g., Villringer et al., 1993; Hoshi, 2011).

Within the human–computer interaction community, NIRS has been primarily used in two ways: (1) for *evaluating* human–machine interactions (e.g., Hirshfield et al., 2009a, 2011a), and more recently, (2) as additional input *to adapt* user interfaces and computer systems based on the user's cognitive state (e.g., Solovey et al., 2012), which is generally referred to as a *passive* brain–computer interface (Zander and Kothe, 2011).

While there are a growing number of EEG-based brain–computer interfaces (BCIs) (e.g., George and Lecuyer, 2010), the development of NIRS-based BCIs has generally lagged behind (e.g., see Table 1 vs. Frey et al., 2014). Moreover, as a consequence of the NIRS literature being dispersed across many publication outlets in HCI, neuroimaging, and brain–computer interface communities (and furthermore, of inconsistencies in results within and between these fields), the efficacy of NIRS-BCIs in realistic human–robot interactions (Canning and Scheutz, 2013) and HCI settings (Strait et al., 2013b) is relatively unknown and unexplored.

**Table 1:**
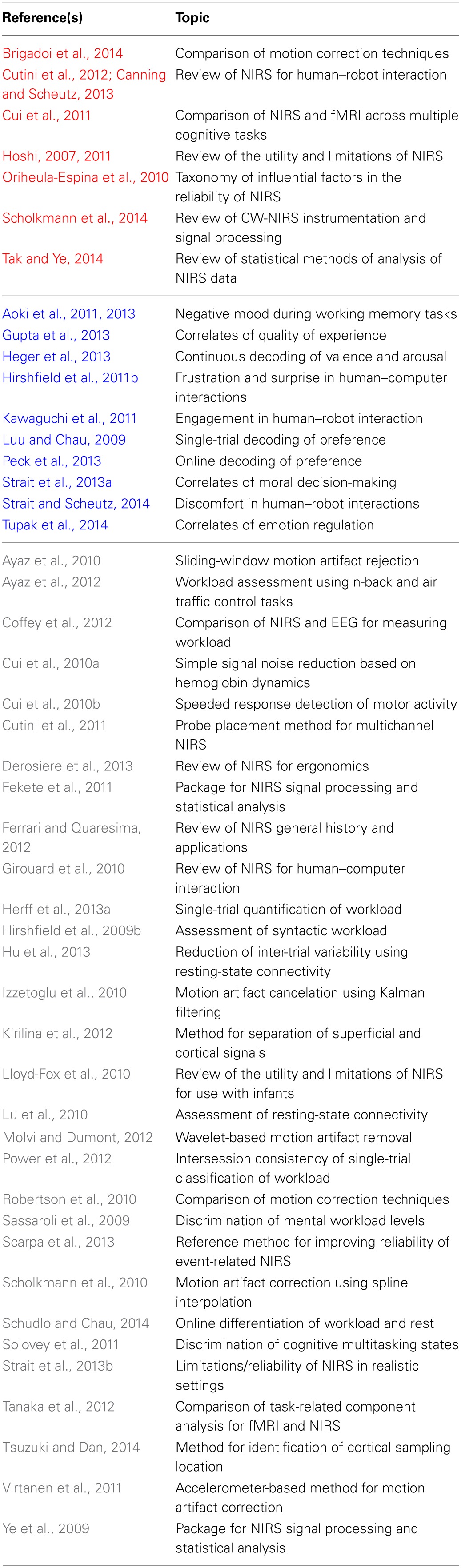
**Resources for and applications of recent NIRS-based systems**.

*In red: useful reviews of NIRS instrumentation and applications. In blue: NIRS based investigations of neural signals that reflect affective states in particular*.

To date, NIRS has been shown to be quite successful in measuring a number of cognitive and affective states (e.g., Cutini et al., 2012) in highly controlled laboratory settings. Yet, substantial challenges persist concerning signal processing for more realistic settings, many of which have already been identified (e.g., Hoshi, 2003, 2007; Plichta et al., 2007; Cutini et al., 2011; Hoshi, 2011; Krusienski et al., 2011; Kirilina et al., 2012; Canning and Scheutz, 2013; Hu et al., 2013; Strait et al., 2013b, 2014b). And while these challenges are not necessarily unique to NIRS, (e.g., see the limitations of using functional magnetic resonance imaging Cacioppo et al., 2003; Logothetis, 2008 and EEG Lotte, 2011; Ohara et al., 2011; Brouwer et al., 2013; Frey et al., 2014), we are still lacking adequate solutions to overcome them.

Hence, the goals of this paper are the following: to provide (1) a review of what can be currently done with NIRS-BCIs for measuring cognitive and affective user states relevant to HCI, (2) a discussion of the effects of naturalistic and unconstrained interaction settings of HCI on signal reliability, and (3) a quantitative comparison of the performance of three modeling approaches in these more realistic settings. We first start with a review of the technology, including an overview of current NIRS-based systems and their limitations. We then identify and evaluate some of the challenges for model reliability, and conclude with a discussion of directions for future research to overcome those challenges.

## 2. Functional near infrared spectroscopy

Functional near infrared spectroscopy is a neuroimaging technique (similar to fMRI) for measuring changes in blood-oxygenation (Hoshi, 2011). Due to the differences in absorptivity between oxygenated and deoxygenated hemoglobin and the transparency of biological tissue to light in the 700–1000 nm range, NIRS is able to capture the hemodynamic changes via the coupling of infrared light emission and detection (Hoshi, 2011). Change in hemoglobin concentration following a precipitating stimulus is referred to as the hemodynamic response (HDR) and can be used to make inferences about functional areas of the brain. Unlike EEG, however, most NIRS-based studies find the onset of the response lags behind the triggering events by at least 1–2 s (e.g., Cui et al., 2011), which then peaks 4–8 s after the stimulus onset and then dips back down over the course of several more seconds as homeostasis is reestablished (e.g., Matthews and Pearlmutter, 2008; Hoshi, 2011). For detailed reviews of hemodynamics and NIRS instrumentation, see for example: Lloyd-Fox et al. (2010); Hoshi (2011); Ferrari and Quaresima (2012); Scholkmann et al. (2014).

### 2.1. Using NIRS to measure cognitive states

Within the field of HCI, discrimination of workload-based states is the predominant application of NIRS (e.g., Nozawa, 2010; Hirshfield et al., 2011a; Ayaz et al., 2012; Coffey et al., 2012; Herff et al., 2013a,b; Schudlo and Chau, 2014). There are also a growing number of affect-related studies using NIRS, with the primary focus on the detection of negatively-valenced and high-arousal states (e.g., Tupak et al., 2014). Table 1 shows a number of relevant NIRS-related publications and a summary of their topics. Additionally, there are several comprehensive reviews of the utility and limitations of NIRS in general (Hoshi, 2011; Cutini et al., 2012; Brigadoi et al., 2014; Tak and Ye, 2014) and for human–robot interaction (Canning and Scheutz, 2013) in particular.

Although this set of measureable states (i.e., workload, negative affect) is a subset of that which is achieved using EEG (i.e., workload, attention, vigilance, fatigue, error recognition, affect, engagement, flow, and immersion; see Frey et al., 2014), NIRS may serve as a complimentary or alternative modality. Specifically, while some comparisons of EEG versus NIRS for workload detection found that NIRS is less effective across a population (i.e., better-than-chance classifications were observed for only 50% of participants using NIRS versus 80% of participants using EEG) (Coffey et al., 2012), NIRS has also been found to achieve better overall discrimination of two levels of workload compared to EEG (Hirshfield et al., 2009a). Hence, a combination of the two (both NIRS and EEG) may be more appropriate for general deployment in workload-related activities.

Moreover, as the prefrontal cortex shows functional coupling in response to emotionally-charged tasks (e.g., Strait et al., 2013a), NIRS may be of greater utility (than EEG) for the detection such localized affect-related brain activity. For instance, recent EEG-based studies have shown recognition rates of only mid-50% for two-way classification (Frey et al., 2014) which is substantially less than what has been achieved in similar paradigms using NIRS which show recognition rates of mid to high 60% (Heger et al., 2013). Although recent EEG-based research shows successful recognition rates of 85–90% for arousal and valenced-states (Liu et al., 2011), artifacts arising from the electrical activity of facial muscles were not controlled for in this work. Given such artifacts are both inherent to emotion induction paradigms and have been shown to have significant effects on frontal EEG channels (e.g., Heger et al., 2011), it is unlikely the above results are reliably detecting brain activity (versus EMG activity of facial muscles). Hence, NIRS may be a useful alternative for measuring affect-related activity. In particular, for NIRS-based affect-related studies (e.g., Aoki et al., 2011, 2013; Hirshfield et al., 2011a; Strait et al., 2013a; Strait and Scheutz, 2014; Tupak et al., 2014), the results are highly consistent across the various efforts and moreover, across a diverse set of contexts (i.e., threat, working memory tasks, moral decision-making, human-robot interactions) in which detection rates significantly better than chance have been achieved. However, as this body of work—similar to Liu et al. (2011)—relies on frontally-situated probes that are proximal to primary facial muscles, the measurements might still reflect some degree of EMG artifacts rather brain activity alone.

Furthermore, as the majority of these studies have been conducted in offline settings, affect detection may still be premature for passive NIRS-BCIs. There exist but a few attempts (moreover, with mixed results) at single-trial and online decoding of affective states (specifically Luu and Chau, 2009; Heger et al., 2013; Peck et al., 2013). Regarding the detection of user preferences (e.g., affinity versus aversion), Luu and Chau originally showed an average classification accuracy of 80% in decoding users' preferences between two possible drinks in a single-trial NIRS paradigm (Luu and Chau, 2009). However, after an issue with the original methodology was identified (Dominguez, 2009), reanalysis yielded an average classification accuracy of 54% (Chau and Damouras, 2009) which was not significantly better than chance. Similarly, in an online classification paradigm, Peck and colleagues investigated preference decoding as a means of providing implicit ratings of movies (Peck et al., 2013). However, comparison of the NIRS-based recommendations (recommendations based on classification of the users' NIRS data) versus random movie recommendations did not show any significant difference. Despite the unsuccessful approaches to decoding of preference states, the work of Heger and colleagues suggests that offline experimentation on the detection of certain affective states may indeed extend to more realistic settings. In Heger et al. (2013), they showed three affect classes (high valence, high arousal, and high valence/arousal) could be reliably (63–69% average classification accuracies) discriminated from neutral for an eight-subject sample in an asynchronous classification paradigm. However, their recognition of high-valenced versus high-arousal states did not perform significantly better than chance (average accuracy of 53%), thus suggesting the granularity of passive NIRS-BCIs for affect recognition is limited.

### 2.2. Exemplars of NIRS-BCIs

While investigation into NIRS-based detection of affect is growing, on the forefront of state-of-the-art NIRS-BCIs is the development of NIRS as a passive input modality (referred to here as “NIRS-pBCI”) based on workload-related user states. Table 2 shows a detailed summary of known demonstrations of NIRS-pBCIs. Aside from the couple aforementioned attempts at online affect detection (Heger et al., 2013; Peck et al., 2013), these systems are primarily based on the decoding of workload-related states (i.e., Matsuyama et al., 2009; Solovey, 2012; Solovey et al., 2012; Girouard et al., 2013; Afergan et al., 2014; Schudlo and Chau, 2014). Here we discuss three such systems in detail regarding their approaches to the online decoding of cognitive states as well as their current limitations.

**Table 2:** **Current passive NIRS-BCI systems (listed by first author)**.

**References**	**Model**	**Latency (s)**	**Classes**	**Accuracy (%)**	***N***
Girouard et al., 2013	Workload	30	2	82	9
Heger et al., 2013	Affect	5	2	68	8
Matsuyama et al., 2009	Workload	9	2	NA	9
Peck et al., 2013	Affect	25	5	27	14
Schudlo and Chau, 2014	Workload	20	2	77	10
Solovey et al., 2012	Workload	40	2	68	3

*Model refers to the type of state information of interest, latency is the delay imposed by the signal processing on onset detection, and N indicates the population sample size*.

#### 2.2.1. Reference channel/thresholding

Matsuyama and colleagues created a simple, proof-of-concept NIRS-pBCI based on the detection of workload-related hemodynamic changes (Matsuyama et al., 2009). Their study was a preliminary attempt at using passive monitoring of users' cognitive state to adapt a robot's behavior. Using a 35-channel NIRS instrument, they measured participants' prefrontal cortex while they solved arithmetic problems. As a proof-of-concept of NIRS-based robot adaptivity, they developed their NIRS-pBCI to send a primitive motion command to a robot when it detected changes in hemoglobin associated with the arithmetic problem solving (i.e., when an increase in oxygenated hemoglobin was observed corresponding to the participant actively working on a arithmetic problem). They used a simple combination of thresholding and reference channel for noise subtraction to detect task-evoked changes in oxy-hemoglobin. Specifically, to avoid noise from widespread brain activity, they computed the difference between two regions—a target region and a reference region (F7-F4, coordinates according to the International 10–20 placement system). Then, using a single threshold (max F7-F4 difference in oxy-hemoglobin), their NIRS-pBCI would cause the robot to move whenever this threshold was surpassed. While there exist many sound BCIs for the direct control of robotic systems (e.g., Canning and Scheutz, 2013), their NIRS-BCI system was not intended to use workload-related activity to directly control a robot. Rather, it served as an effective demonstration that a NIRS-based BCI can passively monitor a person's cognitive workload to initiate behavioral changes in a robot. However, this work also exposed a particular shortcoming of NIRS that is an obstacle for its effectiveness in more realistic scenarios, namely that of onset detection latency (Canning and Scheutz, 2013). Specifically, using their approach to workload monitoring, the time between a participant beginning the arithmetic problem and the transmission of the motor control signal ranged from just few seconds to over 15 s (Matsuyama et al., 2009). As task-related hemodynamic changes in oxygenated hemoglobin occur over several seconds (Coyle et al., 2007), this delay was (and is) somewhat unavoidable due to the inherent hemodynamics; however, recent work has demonstrated vast reductions in temporal delays to onset detection (Cui et al., 2010b), which suggests improvement may be possible.

#### 2.2.2. Temporal dynamics

Similar to Matsuyama et al. (2009), we previously participated in the development of a passive NIRS-BCI aimed at adapting a robot's behavior based on a person's detected multitasking state (Solovey et al., 2012). A two-probe NIRS instrument (with four sources per probe) was used to image participants' prefrontal cortex, while they worked with two simulated robots on a human-robot team task. Here we designed a naive SVM (support vector machine) classification model based on gross temporal dynamics, built by the Sequential Minimal Optimization (SMO) algorithm available in the Weka (Waikato Environment for Knowledge Analysis^1^) library (Hall et al., 2009) and trained using data collected while participants performed a variant of the n-back task. Specifically, the SVM was trained on feature vectors containing every measure of amplitude of both oxy- and deoxy-hemoglobin over the course of a 40 s period of n-back performance. That is, for a device with a sampling rate of 6.25 Hz and a task period of 40 s, a single training example was a vector of 40 s × 6.25 cycles/second × 2 signals (oxy and deoxy) × 2 probes × 4 sources/probe, or 4000 features. This naive approach was a first attempt at capturing temporal patterns over the full time course of a person performing the n-back task. The n-back task, rather than human–robot team task, was used for training in order to avoid potential variations implicit in the team task, but we expected participants to show similar patterns in their NIRS data across both tasks as both induced similar levels of subjectively reported mental stress.

In the human–robot team task, we hypothesized that adapting the level of a robot's autonomy would lead to better task performance and better perceptions of teamwork. Thus, while participants performed the team task, classifications of their mental workload dynamically adapted the autonomy of one of the robots according to the participant's multitasking state. An initial evaluation (Solovey, 2012; Solovey et al., 2012) showed successful task completion was significantly moderated by adaptivity: the dynamic adaptivity of the robot's autonomy improved task performance (82% of participants successfully completed the team task versus a baseline performance rate of 45%). This system was thus a substantial extension of Matsuyama et al. (2009), as it was the first NIRS-BCI to demonstrate effective improvements on a *realistic* task. However, in a recent series of reinvestigations (Strait et al., 2014b) of this system's classification performance, the average classification accuracy on an alternative dataset (of mental arithmetic) was only 54.5% (*SD* = 14.3%) suggesting limited generalizability of the system's signal processing. Additionally, this NIRS-pBCI was found effective (statistically better than chance) for only 10 of 40 participants in this alternative dataset (Strait et al., 2014b), which suggested limited utility for a more realistic population sample (i.e., when *N* = 40 versus *N* = 3 in the initial evaluation). This finding was consistent with one recent investigation (Coffey et al., 2012) which showed better-than-chance NIRS-based classifications for only 5 out of 10 participants on a workload task, but not with another recent investigation (Hirshfield et al., 2009a), which showed the reverse. Hence it remains to-date unclear whether one modality or the other (EEG versus NIRS) is better for measuring workload-related signals, if either, or if it is largely a function of the signal processing methods employed.

#### 2.2.3. Combination temporal/spatiotemporal dynamics

Schudlo and Chau (2014) also developed an online NIRS–BCI which was driven by a mental arithmetic; however, unlike previous NIRS-pBCIs, their system also accommodated an unconstrained rest state. That is, while previous examples of NIRS-pBCIs have been demonstrated to function in online settings (e.g., Matsuyama et al., 2009; Solovey et al., 2012; Girouard et al., 2013), they all employ a synchronous training paradigm, which does not clearly allow the user to remain in an unconstrained resting state for an unfixed length of time. Given this gap in the NIRS-pBCI literature, Schudlo and Chau investigated whether prefrontal activity corresponding to mental arithmetic and unconstrained rest could be differentiated online at a practical accuracy for more realistic BCI use. Here the prefrontal cortex was sampled (using a nine-channel spectrometer) while participants selected letters from an on-screen scanning keyboard via intentionally controlled brain activity (mental arithmetic). To classify the hemodynamic activity, a combination of temporal features (extracted from the NIRS signals) and spatiotemporal features (extracted from dynamic NIRS topograms) were used in a majority vote combination of multiple linear classifiers. The online classification results showed an average accuracy of 77.4% (*SD* = 10.5%), with 8 of the 10 participants showing accuracies significantly above chance. Considering previous results showing significant detection accuracies in less than half of participants (Coffey et al., 2012; Strait et al., 2014b), the findings of Schudlo and Chau's work are particularly promising, and suggest that mental workload, using a more complex classification approach, may indeed be effective at driving a passive NIRS-BCI.

### 2.3. Considerations

The previous section detailed three examples of state-of-the-art passive NIRS-BCIs, which intended to serve both as proof-of-concept demonstrations of NIRS being successfully utilized as a passive input to a computer system, as well as of the challenges to achieving more robust NIRS-pBCIs. While there are numerous factors that contribute to the reliability and robustness of a NIRS-based system (e.g., Oriheula-Espina et al., 2010), we highlight some of the more pressing of these considerations, as well as the differences in signal processing that may contribute to decrements to signal reliability in moving from offline NIRS-based systems to online, passive BCIs.

In the standard, *offline* approaches to signal processing of NIRS data, the signals are short (3–60 s) and heavily filtered *post hoc* (with roughly the following measures)—*detrending* (removal of low frequency signal artifacts and drift), *smoothing* (removal of systemic artifacts such as cardiac pulsations, respiration, and Mayer waves), *motion correction* (reduction of motion artifacts), and *data reduction* (removal of noisy or corrupt trials; averaging over repetitions of a task and/or truncation of the signal to reduce temporal variation; using summary statistics, e.g., area-under-the-curve, percent signal change to represent the overall hemodynamic response) (see Cui et al., 2010a; Oriheula-Espina et al., 2010; Hoshi, 2011; Brigadoi et al., 2014; Scholkmann et al., 2014; Tak and Ye, 2014). Such processing can result in dramatic reductions of signal noise, however, in online, passive settings, signal processing faces substantial challenges (Canning and Scheutz, 2013; Schudlo and Chau, 2014), three of which we detail here.

#### 2.3.1. Onset latency

In moving from offline to fully online, unconstrained, real-time analysis, NIRS-pBCIs suffer a loss in signal processing as well as task information which may result in increased signal noise, and hence, increased unreliability. Specifically, while offline paradigms have known onsets and offsets of the task stimulus, such an oracle is lost in an online, asynchronous scenario. That is, the difficulty in offline processing is primarily to identify whether a trial contains a significant change in hemodynamic activity in response to a particular stimulus. Whereas, in passive (online) systems, not only must we identify whether the signal contains a significant hemodynamic response, but also where such a response begins and terminates. While these fundamental differences in offline versus online protocols is not a new consideration for the signal processing or EEG communities (e.g., Lotte, 2011), they underscore a necessary consideration when transitioning from proof-of-concept (offline) systems to robust online, passive systems that has yet to receive much discussion regarding NIRS-based BCIs. For instance, while both Girouard and colleagues (Solovey, 2012; Girouard et al., 2013) as well as Schudlo and Chau (Schudlo and Chau, 2014) achieved accuracies that were relatively high for online classification of NIRS data with their NIRS-pBCIs, their systems implicitly required delays in the detection of task-related onsets of 20–40 s. Such delays limit the execution of passive NIRS-based adaptivity to only after a significant amount of time has elapsed.

#### 2.3.2. Participant mobility

In addition to the loss of onset/offset oracles, signal noise is also problematic for passive BCI systems. In particular, unrestricted participant mobility can cause motion artifacts which degrade the NIRS signals (e.g., Canning and Scheutz, 2013). These artifacts can be caused by movement of the sensors on the skin, facial expressions, and head orientation (Matthews and Pearlmutter, 2008; Robertson et al., 2010). As techniques for online, asynchronous filtering are limited (e.g., Ayaz et al., 2010; Cui et al., 2010a), other attempts at combating motion artifacts include restricting participant mobility (e.g., using chin rests and mechanical supports, Coyle et al., 2007), which are not particularly suited for *realistic* HCI settings and furthermore, such restrictions on participant mobility significantly reduce the value gained in using NIRS over fMRI. There are, however, a growing number of proposals for real-time motion artifact correction in natural environments, such as the adjustment of the signal based on statistical associations between oxy- and deoxy-hemoglobin values (Cui et al., 2010a), the use of linear quadratic estimation (Izzetoglu et al., 2010), and the use of complimentary physiological measures (Falk et al., 2011).

#### 2.3.3. Task-unrelated activity

Lastly, task-unrelated activity such as resting-state fluctuations (Hoshi, 2011; Hu et al., 2013) or whole brain activity (Matsuyama et al., 2009) can degrade the signal quality. That is, separating task-related from unrelated cortical activity and signal noise can be difficult in some cases (e.g., Kirilina et al., 2012). For example, to separate task-related activity from unrelated whole brain activity, a reference channel outside the cortical region of interest has been used as a method to subtract out the task-unrelated activity (Matsuyama et al., 2009; Lu et al., 2010; Scarpa et al., 2013). This method, however, is impractical when multiple channels are not available (e.g., as was the case in Solovey et al., 2012) and moreover, assuming the reference is neutral (that the activity at the reference region is unrelated to the task-evoked activity), it relies on the quality of the channel placements, which is in itself a challenge for NIRS (Plichta et al., 2007). However, there are a couple of recent proposals for improving the identification of sampling region using probabilistic registration methods of probe placement based on a reference-MRI database (Tsuzuki and Dan, 2014), as well as for separating superficial from cortical signals (Kirilina et al., 2012) and for using resting-state connectivity for reducing inter-trial variability (Hu et al., 2013).

## 3. Investigation

To empirically investigate some of the aforementioned challenges to signal reliability, we collated a large NIRS dataset which we used in the construction of three basic models. The dataset contains (1) 18 training samples of resting versus workload-induced states, during which participant mobility was restricted; (2) 18 training samples (rest versus workload) where mobility was *unrestricted*; and (3) one testing sample of a more realistic task paradigm (i.e., prolonged rest and task periods similar to the human–robot team task in Solovey et al., 2012). Here, we first compare the performance of three basic NIRS models (using 10-fold cross-validation) when trained on data with and without participant movement. Following, we then look at the relative model performances when applied to the more realistic testing sample.

### 3.1. Dataset

To compare the relative performance of three modeling approaches, as well as the effects of unrestricted participant mobility on model performance, we obtained the dataset from Strait et al. (2013b) for further analysis. The dataset contains 40 Tufts University students and staff (18 male; ages 18–45, *M* = 23.4, *S* = 5.8), sampling prefrontal hemodynamic activity (recorded bilaterally using a two-channel ISS OxiplexTS, with a temporal resolution of 6.25 Hz) while participants performed a workload-inducing arithmetic task. All participants were healthy, right-handed, with normal or corrected-to-normal vision, and reported no known history of neurological or psychiatric disorder. To secure the NIRS probes to the participant's forehead, we used a fitted black cap. To minimize signal noise due to ambient light, the room lights were turned off during the recording periods and all stimuli were presented via white text on a black background. Each participant performed two blocks of the workload task (each block comprised of nine trials of arithmetic, nine trials of rest)—one block with their motion restricted (using a zero-gravity chair and verbal instructions to remain motionless) and one with their motion unrestricted (using a simple office chair and verbal instructions to sit naturally). While the trials were each separated by a 30 s fixation cross, here we refer to trial as a sampling period comprised of the participant performing the task or resting only. That is, the trials contained measurements sampled while the participant was actively performing the task or (exclusive) resting.

#### 3.1.1. Signal processing

Prior to analysis, the dataset was first converted using the modified Beer-Lambert Law (MBLL), which yielded a measure of Hb (deoxygenated) and HbO (oxygenated hemoglobin) at each time point for each of two sensors positioned over the left and right prefrontal cortex (PFC), respectively, for a total of four timeseries signals (left Hb, HbO; right Hb, HbO). We then detrended the signals by subtracting out the signal obtained from a low-pass filter (1st degree Savitsky-Golay with a cut-frequency of 0.01 Hz) and smoothed the resulting signals using another low-pass FIR filter (1st degree Savitsky-Golay with a cut-frequency of 0.15 Hz) to reduce the effects of systemic physiological artifacts (namely, cardiac pulsations and respiration). Lastly, we applied a correlation-based signal correction (Cui et al., 2010a) to reduce the effects of motion artifacts. Although all signal processing was applied *post hoc* and offline, online implementations of similar filters have been suggested to be equally effective (Cui et al., 2010a,b).

#### 3.1.2. Modeling

We constructed our models using the nine arithmetic and nine rest training trials (measured under restricted mobility conditions) based on three relatively successful approaches to classifying NIRS data: (1) the reference channel/threshholding approach described in Matsuyama et al. (2009), and the slightly more complex SVM-based approaches of (2) Cui et al. (2010b) and (3) Solovey et al. (2012). Here we implemented the reference channel/thresholding approach put forth by Matsuyama et al. (2009), such that we calculated the difference in oxy-hemoglobin between the two sensors placed bilaterally on the PFC (left PFC—right PFC). This roughly corresponds to the probe placement used in Matsuyama et al. (2009), with the probe measuring the left PFC placed more anterior and medial to the F7 region of interest. To classify the rest versus workload states, this model compares each time point in the left-right oxy-hemoglobin difference against a single baseline value (the average of the max differences during the observed in the resting trials). If the difference at the current time point exceeds the baseline value, the system classifies it as task-evoked activation. To compare more sophisted approaches, we implemented a simple SVM model based on Cui et al. (2010b) which uses four features—the amplitude of left and right oxy/deoxy—and again performs a classification of each timepoint. While this approach is still relatively simple, it capitalizes on the correlations between oxy/deoxy hemodynamics, as well as possible left/right synchronies. Lastly, we compared both approaches with the results of the model described in Solovey (2012) and Solovey et al. (2012), which uses the entire time course of a training sample (see Strait et al., 2014b for details).

### 3.2. Results

The results of the cross-validation are shown in Table 3, where accuracy refers to the overall recognition rate of both classes (rest and task). The results of the Matsuyama thresholding model (Matsuyama et al., 2009) are depicted in the first column section (average time to onset detection, *M*_*on*_, and average classification accuracy, *M*_*acc*(1)_). The middle column section depicts the results of the simple SVM model (Cui et al., 2010b), and the rightmost column section depicts the results of the more complex SVM model^2^ (Solovey et al., 2012). Using the thresholding approach, we found an average task detection latency of 12.6 (±7.6) s across participants (*N* = 40), with individual averages ranging from 3.1 to 27.5 s (see Table 3, left). However, the recognition rate of this model did not perform better than chance (*M* = 46.6%, *SD* = 17.2%). Whereas, both the more complex SVM models performed significantly above chance recognition levels (simple SVM: *M* = 60.5%, *SD* = 15.8%, *p* < 0.0001 and complex SVM: *M* = 54.5%, *SD* = 14.3%, *p* = 0.0037). However, between these two SVMs, the more simple approach of the two (Cui et al., 2010b) performed significantly better both in terms of classification accuracy (*p* = 0.0035) and across the subject population (with 20/40 participants showing significant recognition rates) versus the more complex approach (with 10/40 showing significant rates).

**Table 3:**
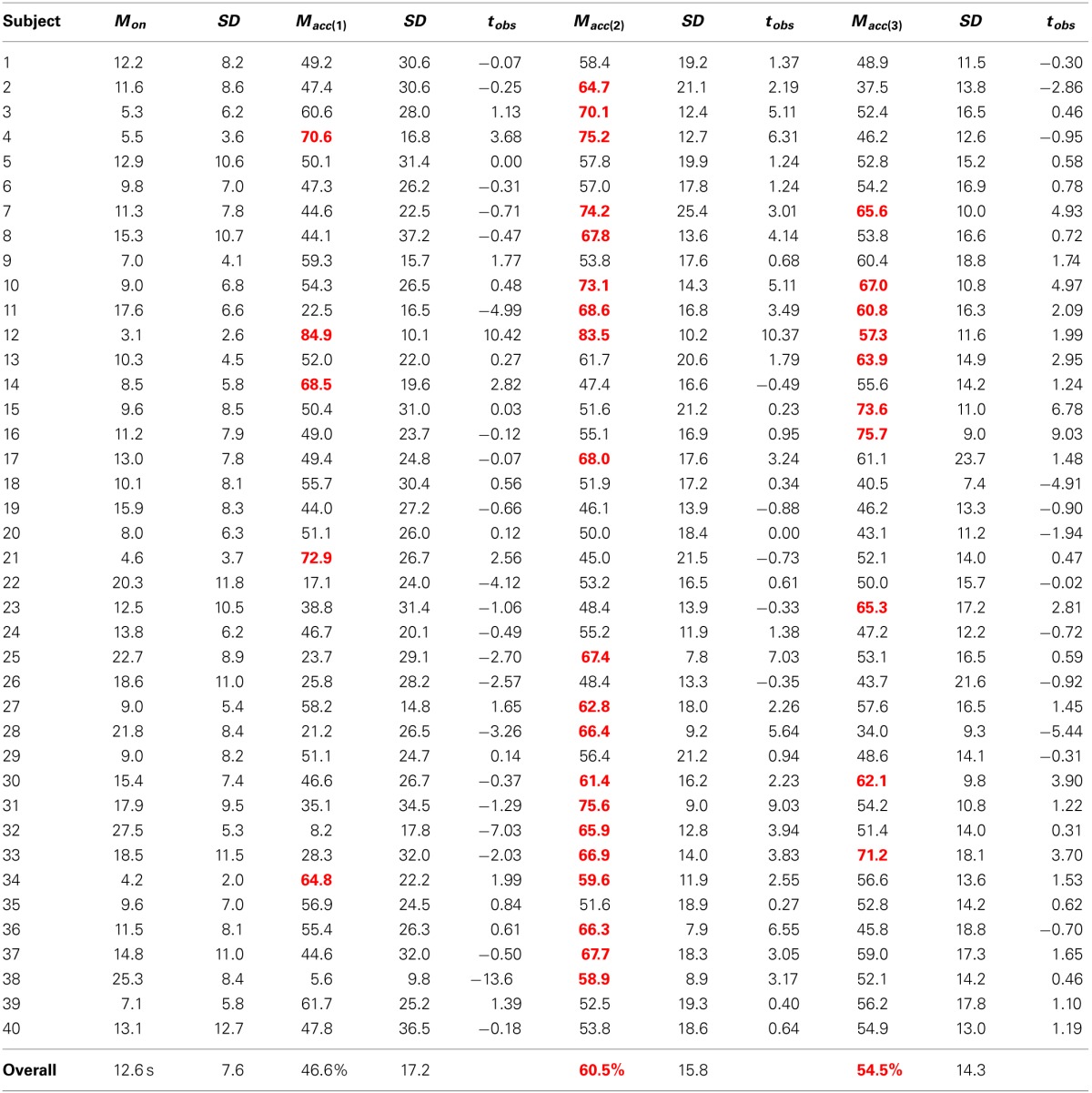
**Relative model performances in nine-fold cross-validation**.

*The Matsuyama approach is shown in the left column section (with both onset latency and classification accuracy shown). Middle shows the model based on Cui et al. and far right, the Solovey et al. model. In red: rates that are significantly above chance (right-tailed t-test, t_crit_(8) = 1.8595)*.

To examine the effects of (semi) unrestricted participant mobility, we next re-constructed each of the three models using the motion-*unrestricted* set of training samples (again nine of each rest and arithmetic trials). Using nine-fold cross-validation of these samples, we found neither the thresholding nor simple SVM approaches were significantly affected in terms of classification accuracy (*M* = 45.2%, *SD* = 18.2%, *p* = 0.5459; and *M* = 60.4%, *SD* = 15.4%, *p* = 0.8850, respectively), nor in onset latency for the thresholding approach (*M* = 13.7 s, *SD* = 5.7 s, *p* = 0.1446). However, the performance of the more complex SVM model was significantly degraded, with an average classification accuracy of 25.3% (*SD* = 7.3%, *p* < 0.0001).

To investigate the relative performances of each of these three models in a more realistic task paradigm, we tested each of the classification approaches (using the models trained on the motion-*restricted* training samples) on the testing sample (3.5 min rest, 3.5 min arithmetic, 3.5 min post-arithmetic rest). Here we observed a significant reduction in classification accuracy for the simple SVM model (*M* = 54.6%, *SD* = 14.4%, *t*_*obs*_ = 1.74), but not the complex SVM (*M* = 48.5%, *SD* = 15.1%, *t*_*obs*_ = 0.67). However, the simple SVM still performed significantly above chance (*t*_*obs*_ = 2.02, *t*_*crit*_(39) = 1.68). There was not any significant change in accuracy for the thresholding model (*M* = 43.9%, *SD* = 10.5%, *t*_*obs*_ = 0.84).

### 3.3. Discussion

#### 3.3.1. Model performance

**Figure 1:**
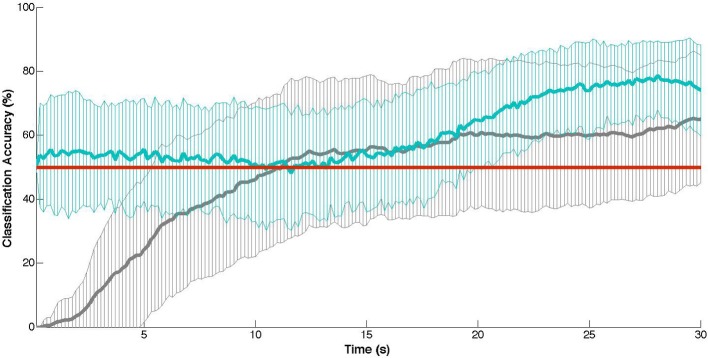
**Cross-validation results: mean classification accuracy (± ***SD***) at each time point of the training task (30 s) with chance-level accuracies indicated in red**. In gray: the thresholding approach (Matsuyama et al., 2009). In blue: the naive SVM approach (Cui et al., 2010b).

In comparison to Matsuyama et al. (2009), the simple reference channel/thresholding combination approach on the dataset used here showed onset latencies substantially slower (*M* = 12.6 s, *SD* = 7.6 s) than theirs (*M* = 9.1 s, *SD* = 4.3 s). This increase in delay and variability may be in part due to a different and larger sample population, as well as the placement of the probes (the positioning used here was inexact and slightly more anterior and medial in comparison to Matsuyama et al., 2009). Hence, the measured activity by the channel used for reference may not have been entirely distinct from the target region-of-interest. In any case, our results confirm a temporal limitation for workload-based state detection, at least when using a minimal (two-probe) NIRS instrument. That is, a fair onset detection delay (9–13 s) will be encountered using this method (see Figure 1). However, more problematic for this method is the classification accuracy: which failed to perform any better than chance overall. While this naive detection approach may work appropriately for contexts in which the duration of the passive adaptivity is not important, for contexts in which it is (e.g., if a robot should only act autonomously while a person is multitasking or mentally stressed), this may not serve as the best model. Similarly, a model that is very complex also may not be the best approach. Specifically, the more simplistic SVM model significantly outperforms the more complex SVM, both in terms of overall accuracy (60.5% versus 54.5%) and within the population (effective for 20 participants versus only 10 using the complex SVM). As SVMs are known to produce poor performance on highly-dimensional data with few training samples (Cortes and Vapnik, 1995), this difference in performance here between the two SVMs might be attributable to the availability of only 18 training samples total in combination with the complex SVM (which employs 4000 features in its model of workload-based activity) versus the simple SVM (which makes use of only four features). For instance, Power and colleagues showed a nearly 15% improvement in classification accuracy in using 80 versus 10 training samples (Power et al., 2012). Thus, given more training samples, we might expect the complex SVM approach to show better recognition rates.

#### 3.3.2. Model performance subject to movement

When we next re-trained our models using the training samples with semi (participants were still tethered within range of the NIRS device) unrestricted participant mobility, we found neither the thresholding nor simple SVM approaches were significantly affected. However, the performance of the more complex SVM model was significantly degraded, with an average classification accuracy of 25.3% (*SD* = 7.3%, *p* < 0.0001). This difference in effects may be due to the difference in approach, where the more simplistic approaches of Matsuyama et al. (2009) and Cui et al. (2010b) classify the NIRS signal at every time point versus the more complex model which classifies a sizable window of the data. Hence, while a motion artifact may significantly degrade the overall measurement sample (thus resulting in lower accuracy of the complex SVM), an individual timepoint may not be so influenced. Potential influences on these models, however, may have been obscured in part by the filtering methods (namely, the correlation-based signal correction to attenuate movement artifacts). Hence it is worth further consideration when developing a NIRS-BCI, as to what signal processing is necessary depending on the context in which it will be used (i.e., if participants will be moving). Lastly, we looked at model performance given a more realistic task paradigm. Here we observed a significant reduction in overall classification accuracy for the simple SVM model, but not for the complex SVM or thresholding model. While the performance of the simple SVM was still statistically significantly above chance, passive adaptivity of a system based on this model would be unlikely to have any serious effects (and thus would be considerably difficult to measure in terms of behavior enhancements of the user).

#### 3.3.3. Limitations

In this section, we systematically investigated three recently-proposed models of NIRS data and their performances when subject to certain factors of more realistic HCI settings (namely participant motion and semi-undefined task durations). While this evaluation serves to highlight the challenges of these factors to achieving more robust NIRS-based systems, there are also a number of limitations to the interpretation of results. In particular, all three modeling approaches performed significantly worse than prior work, with the thresholding approach showing a substantial increase in onset latency and the two SVMs a substantial decrease in accuracy (roughly 15% and 13%, respectively) than the models on which they were based. It is likely that these differences are at least in part due to the sample size, as the sample population used in this study is meaningfully larger than all prior work (*N* = 7 in Matsuyama et al. and *N* = 3 in both Cui et al. and Solovey et al.). It is also likely that they are attributable partially to differences in the task (e.g., numeric versus the alphameric n-back task used in Solovey et al.), region of measurement (prefrontal cortex versus motor cortex measured in Cui et al.), and placement of probes (the 10–20 system was used in Matsuyama et al., but no standardized coordinates were used in this investigation). Hence, it is impossible to speculate as to whether the above effects would be observed in exact replications of prior work. However, these limitations in themselves raise an important consideration regarding NIRS-based research: specifically, whether underpowered studies generalize over larger populations and whether the methods for signal processing and modeling generalize across functional regions of the brain and over a variety of tasks.

## 4. Conclusions

The aim of this paper was to provide (1) an overview of what we can do with NIRS-BCIs for measuring cognitive and affective user states, (2) a discussion of the effects of naturalistic and unconstrained interaction settings of HCI on signal reliability, and (3) a quantitative comparison of the performance of three recent modeling approaches in these more realistic settings. Specifically, we described two primary cognitive and affective states (mental workload and negative affect) measureable with NIRS, as well as two modes of use (evaluatory and passive). Additionally, we emphasized the distinction of offline versus online (real-time) signal processing for NIRS-based BCIs. The prototypical application of NIRS as an evaluation tool is as an offline *post hoc* analysis of a signal recorded during some stimulus. However, the usage of NIRS as a passive BCI (involving the online processing of hemodynamic data) has emerged, and with it, a number of challenges have followed.

We discussed some of those key challenges (participant mobility, more naturalistic interaction) and investigated their effects with a comparative analysis of three recently-proposed modeling techniques. The results of our investigation highlight several considerations, including detection latencies (the temporal delay between a precipitating stimulus and the detection of the stimulus-evoked hemodynamic changes), performance of the model in more naturalistic contexts (i.e., when participant mobility is unrestricted), and the generalizability of current training paradigms (i.e., offline, time-restricted) to the asynchronous, online paradigms of more realistic settings (e.g., Brouwer et al., 2013). The results also underscore several additional considerations, namely efficacy of a NIRS-BCI across a population (i.e., whether the signal processing and modeling approach effective for the whole population or only a small proportion) and task/region-specificity of a technique. While these challenges are not particularly new to the field, or to BCI in general, both the review of the literature and the empirical evaluation highlight the dependencies between performance, signal processing, and experimental context. Research efforts on all these fronts are mutually complementary and necessary to the advancement of NIRS as a tool for human–computer interaction.

NIRS-based systems have already been used in a range of applications, such as the quantification of mental workload and differentiation of aroused/valenced states; however, substantial challenges remain to be addressed before NIRS can become a practical and robust tool for passive BCIs. The challenges emphasized here concern detection latency, signal processing, as well as better understanding of hemodynamic changes over undefined task durations. While there are numerous challenges that have been raised previously (both in NIRS and EEG research), they remain to-date unaddressed. It is thus our hope that this survey and dataset will facilitate researchers to actively engage in NIRS-related research that will help overcome current challenges and make NIRS a more robust and useful tool to the BCI community.

### Conflict of interest statement

The authors declare that the research was conducted in the absence of any commercial or financial relationships that could be construed as a potential conflict of interest.

## Footnotes

^1^The Weka Java libarary contains a collection of common tools for data processing, classification, visualization, and other common analyses for data mining. For more information, see Hall et al. (2009).

^2^The model based on Solovey et al. (2012) and results of its cross-validation are also described in Strait et al. (2014b). However, all additional analyses and discussion presented here of its performance are novel.

## References

[B1] Afergan D. Peck E. Solovey E. Jenkins A. Hincks S. Chang R. (2014). Dynamic difficulty using brain metrics of workload, in CHI. New York, NY: ACM

[B2] Aoki R. Sato H. Katura T. Matsuda R. Koizumi H. (2013). Correlation between prefrontal cortex activity during working memory tasks and natural mood independent of personality effects: an optical topography study. Psychiatry Res. Neuroimag. 212, 79–87 10.1016/j.pscychresns.2012.10.009 23489672

[B3] Aoki R. Sato H. Katura T. Utsugi K. Koizumi H. Matsuda R. (2011). Relationship of negative mood with prefrontal cortex activity during working memory tasks: an optical topography study. Neurosci. Res. 70, 189–196 10.1016/j.neures.2011.02.011 21382424

[B4] Ayaz H. Izzetoglu M. Shewokis P. Onaral B. (2010). Sliding-window motion artifact rejection for functional near-infrared spectroscopy. Conf. Proc. IEEE Eng. Med. Biol. Soc. 2010, 6567–6570 10.1109/IEMBS.2010.5627113 21096508

[B5] Ayaz H. Shewokis P. Bunce S. Izzetoglu K. Willems B. Onaral B. (2012). Optical brain monitoring for operator training and mental workload assessment. Neuroimage 59, 36–47 10.1016/j.neuroimage.2011.06.023 21722738

[B6] Bainbridge W. A. Nozawa S. Ueda R. Okada K. Inaba M. (2012). A methodological outline and utility assessment of sensor-based biosignal measurement in human-robot interaction. Int. J. Soc. Rob. 4, 303–316 10.1007/s12369-012-0146-y

[B8] Brigadoi S. Ceccherini L. Cutini S. Scarpa F. Scatturin P. Selb J. (2014). Motion artifacts in functional near-infrared spectroscopy: a comparison of motion correction techniques applied to real cognitive data. Neuroimage 85, 181–191 10.1016/j.neuroimage.2013.04.082 23639260 PMC3762942

[B9] Brouwer A.-M. van Erp J. Heylen D. Jensen O. Poel M. (2013). Effortless passive BCIs for healthy users, in *HCII*, (Berlin, Heidelberg: Springer), 615–622

[B10] Cacioppo J. Berntson G. Lorig T. Norris C. Rickett E. Nushbaum H. (2003). Just because you're imaging the brain doesn't mean you can stop using your head: a primer and set of first principles. J. Personality Soc. Psychol. 85, 650–661 10.1037/0022-3514.85.4.650 14561119

[B11] Canning C. Scheutz M. (2013). Function near-infrared spectroscopy in human-robot interaction. J. Hum. Rob. Interact. 2, 62–84 10.5898/jhri.v2i3.144

[B12] Chau T. Damouras S. (2009). Reply to ‘on the risk of extracting relevant information from random data.’ J. Neural Eng. 6, 058002 10.1088/1741-2560/6/5/058002 19667460

[B13] Coffey E. Brouwer A.-M. van Erp J. (2012). Measuring workload using a combination of electroencephalography and near infrared spectroscopy, in *HFES* 10.1177/1071181312561367

[B14] Cortes C. Vapnik V. (1995). Support-vector networks. Mach. Learn. 20, 273–297 17306960

[B15] Coyle S. Ward T. Markham C. (2007). Brain-computer interface using a simplified functional near-infrared spectroscopy system. J. Neural Eng. 4, 219 10.1088/1741-2560/4/3/007 17873424

[B16] Cui X. Bray S. Bryant D. Glover G. Reiss A. (2011). A quantitative comparison of nirs and fmri across multiple cognitive tasks. Neuroimage 54, 2808–2821 10.1016/j.neuroimage.2010.10.069 21047559 PMC3021967

[B17] Cui X. Bray S. Reiss A. (2010a). Functional near infrared spectroscopy (NIRS) signal improvement based on negative correlation between oxygenated and deoxygenated hemoglobin dynamics. Neuroimage 49, 3039–3046 10.1016/j.neuroimage.2009.11.050 19945536 PMC2818571

[B17b] Cui X. Bray S. Reiss A. (2010b). Speeded near infrared spectroscopy (NIRS) response detection. PLoS ONE 5:e15474 10.1371/journal.pone.0015474 21085607 PMC2978722

[B19] Cutini S. Moro S. Bisconti S. (2012). Functional near infrared optical imaging in cognitive neuroscience: an introductory review. J. Near Infrared Spectrosc. 20, 75–92 10.1255/jnirs.969

[B20] Cutini S. Scatturin P. Zorzi M. (2011). A new method based on ICBM152 head surface for probe placement in multichannel fNIRS. Neuroimage 54, 919–927 10.1016/j.neuroimage.2010.09.030 20851195

[B21] Derosiere G. Mandrick K. Dray G. Ward T. Perrey S. (2013). NIRS-measured prefrontal cortex activity in neuroergonomics: strengths and weaknesses. Front. Hum. Neurosci. 7:583 10.3389/fnhum.2013.00583 24065906 PMC3777133

[B22] Dominguez L. (2009). On the risk of extracting relevant information from random data. J. Neural Eng. 6, 058001 10.1088/1741-2560/6/5/058001 19667460

[B23] Falk T. Guirgis M. Power S. Chau T. (2011). Taking nirs-bcis outside the lab: achieving robustness against environment noise. Neural Syst. Rehabil. Eng. 19, 136–146 10.1109/TNSRE.2010.2078516 20876031

[B24] Fekete T. Rubin D. Carlson J. Mujica-Parodi L. (2011). The NIRS analysis package: noise reduction and statistical inference. PLoS ONE 6:e24322 10.1371/journal.pone.0024322 21912687 PMC3166314

[B25] Ferrari M. Quaresima V. (2012). A brief review on the history of human functional near-infrared spectroscopy (fNIRS) development and fields of application. Neuroimage 63, 921–935 10.1016/j.neuroimage.2012.03.049 22510258

[B26] Frey J. Mühl C. Lotte F. Hachet M. (2014). Review of the use of electroencephalography as an evaluation method for human-computer interaction, in International Conference on Physiological Computing Systems (PhyCS). (Lisbon).

[B27] George L. Lecuyer A. (2010). An overview of research on ‘passive’ brain-computer interfaces for implicit human-computer interaction in International Conference on Applied Bionics and Biomechanics. (Venice).

[B28] Girouard A. Solovey E. Hirshfield L. Peck E. Chauncey K. Sassaroli A. (2010). From brain signals to adaptive interfaces: using fNIRS in HCI, in Brain-Computer Interfaces, eds Tan D. S. Nijholt A. (New York, NY: Springer), 221–237

[B29] Girouard A. Solovey E. Jacob R. (2013). Designing a passive brain computer interface using real time classification of functional near-infrared spectroscopy. Int. J. Auton. Adapt. Commun. Syst. 6, 26–44 10.1504/IJAACS.2013.050689

[B30] Gupta R. Laghari K. Arndt S. Schleicher R. Moller S. O'Shaughnessy D. (2013). Using fNIRS to characterize human perception of TTS system quality, comprehension, and fluency: preliminary findings, in International Workshop on Perceptual Quality of Systems (PQS).

[B31] Hall M. Frank E. Holmes G. Pfahringer B. Reutemann P. Witten I. (2009). The WEKA data mining software: an update. ACM SIGKDD Explor. Newslett. 11, 10–18 10.1145/1656274.1656278

[B32] Heger D. Mutter R. Herff C. Putze F. Schultz T. (2013). Continuous recognition of affective states by functional near infrared spectroscopy signals, in Affective Computing and Intelligent Interaction (ACII). 10.1109/ACII.2013.156

[B33] Heger D. Putze F. Schultz T. (2011). Online recognition of facial actions for natural EEG-based BCI applications, in Affective Computing and Intelligent Interaction (ACII). (Berlin, Heidelberg: Springer-Verlag), 436–446

[B34] Herff C. Heger D. Fortmann O. Hennrich J. Putze F. Schultz T. (2013a). Mental workload during n-back task—quantified in the prefrontal cortex using fNIRS. Front. Hum. Neurosci. 7:935 10.3389/fnhum.2013.00935 24474913 PMC3893598

[B35] Herff C. Heger D. Putze F. Hennrich J. Fortmann O. Schultz T. (2013b). Classification of mental tasks in the prefrontal cortex using fNIRS. Conf. Proc. IEEE Eng. Med. Biol. Soc. 2013, 2160–2163 10.1109/EMBC.2013.6609962 24110149

[B36] Hirshfield L. Chauncey K. Gulotta R. Girouard A. Solovey E. Jacob R. (2009a). Combining electroencephalograph and functional near infrared spectroscopy to explore users' mental workload, in HCII, (Heidelberg: Springer-Verlag Berlin), 239–247

[B37] Hirshfield L. Solovey E. Girouard A. Kebinger J. Jacob R. Sassaroli A. (2009b). Brain measurement for usability testing and adaptive interfaces: an example of syntactic workload with functional near infrared spectroscopy, in CHI, (New York, NY: ACM), 2185–2194

[B38] Hirshfield L. Gulotta R. Hirshfield S. Hincks S. Russell M. Ward R. (2011a). This is your brain on interfaces: enhancing usability testing with functional near-infrared spectroscopy, in CHI, (New York, NY: ACM), 373–382

[B39] Hirshfield L. Hirshfield S. Hincks S. Russell M. Ward R. Williams T. (2011b). Trust in human-computer interactions as measured by frustration, surprise, and workload, in Foundations of Augmented Cognition (Orlando, FL), 507–516

[B40] Hoshi Y. (2003). Functional near-infrared optical imaging: utility and limitations in human brain mapping. Psychophysiology 40, 511–520 10.1111/1469-8986.00053 14570159

[B41] Hoshi Y. (2007). Functional near-infrared spectroscopy: current status and future prospects. J. Biomed. Opt. 12:062106 10.1117/1.2804911 18163809

[B42] Hoshi Y. (2011). Towards the next generation of near-infrared spectroscopy. Philos. Trans. R. Soc. A Math. Phys. Eng. Sci. 369, 4425–4439 10.1098/rsta.2011.0262 22006899

[B44] Hu X. Hong K. Ge S. (2013). Reduction of trial-to-trial variability in functional near-infrared spectroscopy signals by accounting for resting-state functional connectivity. J. Biomed. Opt. 18:17003 10.1117/1.JBO.18.1.017003 23291618

[B45] Izzetoglu M. Chitrapu P. Bunce S. Onaral B. (2010). Motion artifact cancellation in NIR spectroscopy using discrete kalman filtering. Biomed. Eng. Online 9, 16 10.1186/1475-925X-9-16 20214809 PMC2846950

[B46] Kawaguchi Y. Wada K. Okamoto M. Tsujii T. Shibata T. Sakatani K. (2011). Investigation of brain activity after interaction with seal robot measured by fnirs, in Robot and Human Interactive Communication, 308–313 10.1109/ROMAN.2012.6343812

[B47] Kirilina E. Jelzow A. Heine A. Niessing M. Wabnitz H. Bruhl R. (2012). The physiological origin of task-evoked systemic artifacts in functional near infrared spectroscopy. Neuroimage 61, 70–81 10.1016/j.neuroimage.2012.02.074 22426347 PMC3348501

[B48] Krusienski D. Grosse-Wentrup M. Galan F. Coyle D. Miller K. Forney E. (2011). Critical issues in state-of-the-art brain-computer interface signal processing. J. Neural Eng. 8:025002 10.1088/1741-2560/8/2/025002 21436519 PMC3412170

[B49] Liu Y. Sourina O. Nguyen M. (2011). Real-time EEG-based emotion recognition and its applications, in Transactions on Computational Science XII, eds Gavrilova M. L. Kenneth Tan C. J. Sourin A, Sourina O, (Berlin: Springer), 256–277 10.1007/978-3-642-22336-5_13

[B50] Lloyd-Fox S. Blasi A. Elwell C. (2010). Illuminating the developing brain: the past, present and future of functional near infrared spectroscopy. Neurosci. Biobehav. Rev. 34, 269–284 10.1016/j.neubiorev.2009.07.008 19632270

[B51] Logothetis N. (2008). What we can do and what we cannot do with fMRI. Nature 453, 869–878 10.1038/nature06976 18548064

[B52] Lotte F. (2011). Brain-computer interfaces for 3d games: hype or hope? in Foundations of Digital Games (New York, NY: ACM), 325–327 10.1145/2159365.2159427

[B53] Lu C. Zhang Y. Biswal B. Zang Y. Peng D. Zhu C. (2010). Use of fNIRS to assess resting state functional connectivity. J. Neurosci. Methods 186, 242–249 10.1016/j.jneumeth.2009.11.010 19931310

[B54] Luu S. Chau T. (2009). Decoding subjective preference from single-trial near-infrared spectroscopy signals. J. Neural Eng. 6, 016003 10.1088/1741-2560/6/1/016003 19104138

[B55] Matsuyama H. Asama H. Otake M. (2009). Design of differential near-infrared spectroscopy based brain machine interface, in Robot and Human Interactive Communication, 775–780 10.1109/ROMAN.2009.5326215

[B56] Matthews F. Pearlmutter B. (2008). Hemodynamics for brain-computer interfaces. Signal Process. 25, 87–94 10.1109/MSP.2008.4408445

[B57] Molvi B. Dumont G. (2012). Wavelet-based motion artifact removal for functional near-infrared spectroscopy. Physiol. Meas. 33, 259 10.1088/0967-3334/33/2/259 22273765

[B58] Nozawa T. (2010). Autonomous adaptive agent with intrinsic motivation for sustainable hai. J. Intell. Learn. Syst. Appl. 2, 167–178 10.4236/jilsa.2010.24020

[B59] Ohara K. Sellen A. Harper R. (2011). Embodiment in brain-computer interaction, in CHI. 10.1145/1978942.1978994

[B60] Oriheula-Espina F. Leff D. James D. Darzi A. Yang G. (2010). Quality control and assurance in functional near infrared spectroscopy (fNIRS) experimentation. Phys. Med. Biol. 55, 3701–3724 10.1088/0031-9155/55/13/009 20530852

[B61] Peck E. Afergan D. Jacob R. (2013). Brain sensing as input to information filtering systems, in 4th Augmented Human International Conference, AH'13, (Stuttgart, Germany) 142–149

[B62] Plichta M. Herrmann M. Baehne C. Ehlis A. Richter M. Pauli P. (2007). Event-related functional near-infrared spectroscopy (fNIRS) based on craniocerebral correlations: reproducibility of activation? Hum. Brain Mapp. 28, 733–741 10.1002/hbm.20303 17080439 PMC6871457

[B63] Power S. Kushki A. Chau T. (2012). Intersession consistency of single-trial classification of prefrontal response to mental arithmetic and no-control state by NIRS. PLoS ONE 7:e37791 10.1371/journal.pone.0037791 22844390 PMC3402505

[B64] Robertson F. C. Douglas T. S. Meintjes E. M. (2010). Motion artifact removal for functional near infrared spectroscopy: a comparison of methods. IEEE Trans. Biomed. Eng. 57, 1377–1387 10.1109/TBME.2009.2038667 20172809

[B65] Sassaroli A. Zheng F. Hirshfield L. Coutts M. Girouard A. Solovey E. (2009). Application of near-infrared spectroscopy for discrimination of mental workloads, SPIE. 10.1117/12.807737

[B66] Scarpa F. Brigadoi S. Cutini S. Scatturin P. Zorzi M. Dell'Acqua R. (2013). A reference-channel based methodology to improve estimation of event related hemodynamic response from fNIRS measurements. Neuroimage. 72, 106–119 10.1016/j.neuroimage.2013.01.021 23357074

[B67] Scholkmann F. Kleiser S. Metz A. Zimmermann R. Pavi J. Wolf U. (2014). A review on continuous wave functional near-infrared spectroscopy and imaging instrumentation and methodology. Neuroimage 85, 6–27 10.1016/j.neuroimage.2013.05.004 23684868

[B68] Scholkmann F. Spichtig S. Muehlemann T. Wolf M. (2010). How to detect and reduce movement artifacts in near-infrared imaging using moving standard deviation and spline interpolation. Physiol. Meas. 31, 649–662 10.1088/0967-3334/31/5/004 20308772

[B69] Schudlo L. Chau T. (2014). Dynamic topographical pattern classification of multichannel prefrontal NIRS signals: online differentiation of mental arithmetic and rest. J. Neural Eng. 11:016003 10.1088/1741-2560/11/1/016003 24311057

[B70] Solovey E. Lalooses F. Chauncey K. Weaver D. Parasi M. Scheutz M. (2011). Sensing cognitive multitasking for a brain-based adaptive user interface, in Conference on Human Factors in Computing Systems (CHI), 383–392 10.1145/1978942.1978997

[B71] Solovey E. Schermerhorn P. Scheutz M. Sassaroli A. Fantini S. Jacob R. (2012). Brainput: enhancing interactive systems with streaming fNIRS brain input, in CHI, (New York, NY: ACM), 2193–2202 10.1145/2207676.2208372

[B71b] Solovey E. T. (2012). Real-Time fNIRS Brain Input for Enhancing Interactive Systems. PhD thesis, Tufts University.

[B73] Strait M. Briggs G. Scheutz M. (2013a). Some correlates of agency ascription and emotional value and their effects on decision- making, in Conference on Affective Computing and Intelligent Interaction (ACII), (Washington, DC), 505–510 10.1109/ACII.2013.89

[B74] Strait M. Canning C. Scheutz M. (2013b). Limitations of NIRS-based BCI for realistic applications in human-computer interaction, in BCI Meeting, (Monterey, CA), 6–7

[B75] Strait M. Canning C. Scheutz M. (2014a). Let me tell you! investigating the effects of robot communication strategies in advice- giving situations based on robot appearance, interaction modality and distance, in Human-Robot Interaction (HRI), (New York, NY: ACM). 10.1145/2559636.2559670

[B76] Strait M. Canning C. Scheutz S. (2014b). Reliability of NIRS-Based BCIs: a placebo-controlled replication and reanalysis of Brainput, in Human Factors in Computing (CHI), Extended Abstracts. 10.1145/2559206.2578866

[B77] Strait M. Scheutz M. (2014). Using near infrared spectroscopy to index temporal changes in affect in realistic human–robot interactions, in Physiological Computing Systems (PhyCS), Special Session on Affect Recogntion from Physiological Data for Social Robots.

[B78] Szafir D. Mutlu B. (2012). Pay attention! designing adaptive agents that monitor and improve user engagement, in Conference on Human Factors in Computing Systems (CHI), (New York, NY: ACM), 11–20 10.1145/2207676.2207679

[B79] Tak S. Ye J. (2014). Statistical analysis of fNIRS data: a comprehensive review. Neuroimage 85, 92–103 10.1016/j.neuroimage.2013.06.016 23774396

[B80] Tan D. Nijholt A. editors (2010). Brain-Computer Interfaces: Applying Our Minds to Human-Computer Interaction. London: Springer 10.1007/978-1-84996-272-8

[B81] Tanaka H. Katura T. Sato H. (2012). Task-related component analysis for functional neuroimaging and application to near-infrared spectroscopy data. Neuroimage 64, 308–327 10.1016/j.neuroimage.2012.08.044 22922468

[B82] Tsuzuki D. Dan I. (2014). Spatial registration for functional near-infrared spectroscopy: from channel position on the scalp to cortical location in individual and group analyses. Neuroimage 85, 92–103 10.1016/j.neuroimage.2013.07.025 23891905

[B83] Tupak S. Dresler T. Guhn A. Ehlis A. Fallgatter A. Pauli P. (2014). Implicit emotion regulation in the presence of threat: neural and autonomic correlates. Neuroimage 85, 372–379 10.1016/j.neuroimage.2013.09.066 24096027

[B84] Villringer A. Planck J. Hock C. Schleinkofer L. Dirnagl U. (1993). Near infrared spectroscopy (NIRS): a new tool to study hemodynamic changes during activation of brain function in human adults. Neurosci. Lett. 154, 101–104 10.1016/0304-3940(93)90181-J 8361619

[B85] Virtanen J. Noponen T. Kotilahti K. Virtanen J. Ilmoniemi R. (2011). Accelerometer-based method for correcting signal baseline changes caused by motion artifacts in medical near-infrared spectroscopy. J. Biomed. Opt. 16, 087005 10.1117/1.3606576 21895332

[B86] Ye J. Tak S. Jang K. Jung J. Jang J. (2009). NIRS-SPM: statistical parametric mapping for near-infrared spectroscopy. Neuroimage 44, 428–447 10.1016/j.neuroimage.2008.08.036 18848897

[B87] Zander T. Kothe C. (2011). Towards passive brain-computer interfaces: applying brain-computer interface technology to human-machine systems in general. J. Neural Eng. 8, 025005 10.1088/1741-2560/8/2/025005 21436512

